# Association between polymorphisms in sex hormones synthesis and metabolism and prostate cancer aggressiveness

**DOI:** 10.1371/journal.pone.0185447

**Published:** 2017-10-05

**Authors:** Inmaculada Robles-Fernandez, Luis Javier Martinez-Gonzalez, Manrique Pascual-Geler, Jose Manuel Cozar, Ignacio Puche-Sanz, Maria Jose Serrano, Jose Antonio Lorente, Maria Jesus Alvarez-Cubero

**Affiliations:** 1 GENYO. Centre for Genomics and Oncological Research: Pfizer / University of Granada /Andalusian Regional Government. Granada, Spain; 2 Urology Department, University Hospital Virgen de las Nieves. Granada, Spain; 3 Integral Oncology Division, Clinical University Hospital. Granada, Spain; 4 Laboratory of Genetic Identification, University of Granada–Department of Legal Medicine—Faculty of Medicine, Granada, Spain; 5 Cell Biology Department, University of Granada- Faculty of Sciences, Granada, Spain; National Health Research Institutes, TAIWAN

## Abstract

Novel biomarkers for prostate cancer (PCa) diagnosis and prognosis are necessary to improve the accuracy of current ones employed in clinic. We performed a retrospective study between the association of several polymorphisms in the main genes involved in the synthesis and metabolism of sex hormones and PCa risk and aggressiveness. A total of 311 Caucasian men (155 controls and 156 patients) were genotyped for 9 SNPs in *AR*, *CYP17A1*, *LHCGR*, *ESR1* and *ESR2* genes. Diagnostic PSA serum levels, Gleason score, tumor stage, D´Amico risk and data of clinical progression were obtained for patients at the moment of the diagnosis and after 54 months of follow-up. Chi-squared test were used for comparisons between clinical variables groups, logistic regression for clinical variables associations between SNPs; and Kaplan–Meier for the association between SNPs and time to biochemical progression. We found 5 variants (*CYP17A*1) rs743572, rs6162, rs6163; (*LHCGR*) rs2293275 and (*ESR2*) rs1256049 that were statistically significant according to clinical variables (PSA, D´Amico risk and T stage) on a case-case analysis. Moreover, the presence of *A* and *G* alleles in rs743572 and rs6162 respectively, increase the risk of higher PSA levels (>10 ng/μl). With respect to D´Amico risk rs743572 (*AG-GG*), rs6162 (*AG-AA*) and rs6163 (*AC-AA*) were associated with an increased risk; and last, *AC* and *AA* genotypes for rs6163 were associated with a shorter biochemical recurrence free survival (BRFS) in patients with radical prostatectomy. In multigene analysis, several variants in SNPs rs2293275, rs6152, rs1062577, rs6162, rs6163, rs1256049 and rs1004467 were described to be associated with a more aggressiveness in patients. However, none of the selected SNPs show significant values between patients and controls. In conclusion, this study identified inherited variants in genes *CYP17A1*, *LHCGR* and *ESR2* related to more aggressiveness and/or a poor progression of the disease. According to this study, new promise PCa biomarkers for clinical management could be included in these previous SNPs.

## Introduction

Prostate cancer (PCa) is one of the most prevalent cancers diagnosed in men with 1.1 million cases worldwide in 2012 [1]. However, few conclusive studies have been performed with regard to the genetics of this cancer. One of the main challenges is to find new specific biomarkers that allow clinicians to detect the disease at an early stage, refine risk stratification, and control the course of patients.

So far, only clinical risk classifications, such as that of D´Amico [2], have gained enough evidence to be implemented in routine practice. However, a limitation of these classifications is the lack of integration with other known risk factors and genomic data, which could provide a more personalized risk assessment. Although a number of genomic classifications, such as *FGFR1*, *CDKN1A* and *PMP22* genes [3], have demonstrated their ability to differentiate between low and high-risk patients [3–5], none of them are currently adopted in routine clinical practice [6]. In the same way, there are not genetic biomarkers in the clinical to predict the outcome to different treatments used to attend PCa patients [7].

Androgens play a pivotal role in the development and function of prostate as well as in the pathogenesis and progression of PCa [8]. Moreover, experimental and epidemiological data have suggested that also estrogen signaling may contribute to PCa development and progression [9]. Although recent studies have evaluated polymorphisms in sex hormone metabolism genes, such as *AR*, *SRD5A2*, *CYP17A1* or *ESR* as PCa risk factors [10–12] they did not provide conclusive results.

The aim of the present study was to analyze several SNPs of genes related to the synthesis and metabolism of steroid hormones, such as *CYP17A1* gene (rs743572, rs6162, rs6163 and rs1004467); luteinizing hormone chorionic gonadotropin hormone receptor (*LHCGR*) gene (rs2293275); androgen receptor (*AR*) gene (rs6152 and rs9332696); and estrogen receptor (*ESR1* and *ESR2*) genes (rs1062577 and rs1256049, respectively). We designed a genetic analysis in these 9 SNPs in order to find out genetic associations with PCa risk, biochemical recurrence and/or clinical stratification.

## Materials and methods

### Patients

From 2012 to 2014, a total of 311 subjects with PSA levels ≥4.0 ng/mL meeting the criteria for a prostate biopsy were included in this study (Table 1). Patients with positive biopsy were analyzed for T stage, serum PSA, Gleason score and were classified according to D´Amico risk classification (low, intermediate and high risk). Negative biopsy individuals were considered as controls. All individuals underwent a systematic 20-core ultrasound guided biopsy in order to limit the false negative rate. After primary therapy PSA was monitored every 3 or 6 months to evaluate the existence of biochemical recurrence. All subjects of the study provided a written informed consent to be enrolled, which was previously approved by the Research Ethics Committee of Granada Center (CEI-Granada) following Helsinki ethical declaration.

**Table 1:** Summary of clinical variables.

	Patients n = 156		Controls n = 155	
	n	%	n	%
**Initial PSA (ng/ml)**				
> 4 ≤ 10	84	(53.8)	106	(68.4)
> 10 ≤ 20	33	(21.2)	47	(30.3)
> 20	39	(25)	2	(1.3)
**Gleason Score**				
≤ 7	132	(84.6)	n.a.	
8–10	24	(15.4)	n.a.	
**T Stage**				
T 1–2	137	(87.8)	n.a.	
T 3–4	10	(6.4)	n.a.	
Missing	9	(5.8)		
**D’Amico Risk Group**				
Low	54	(34.6)	n.a.	
Medium	54	(34.6)	n.a.	
High	45	(28.8)	n.a.	
Missing	3	(1.9)		
**Observation Period**				
Median (months)	34.18		n.a.	
Range (months)	1–54		n.a.	
Missing	8		n.a.	

Classification of patients was made following the EAU guidelines on PCa. All subjects included in the study were Caucasian, specifically Iberian. n: total numbers of samples; n.a.: not applicable.

### SNPs selection and genotyping

Immediately before the biopsy, a peripheral blood sample was extracted from patients, put into EDTA coated tubes and stored at -20^0^ C until genomic DNA extraction. A standard organic extraction procedure by phenol/chloroform/isoamyl alcohol and proteinase K, followed by purification with Microcon H 100 filters (Millipore, Germany) was used. To determine extracted DNA purity and concentration a NanoDrop 2000c (Thermo Scientific, USA) was used. Thereafter, DNA was stored at -20^0^ C until genotyping. Five genes involved both directly or indirectly, in androgen synthesis and/or its metabolism were selected: *CYPI7A1*, *AR*, *LHCGR*, *ESR1* and *ESR2* and the picked SNPs were rs6162, rs743572, rs6163 and rs1004467 for *CYPI7A1*; rs6152 and rs9332969 for *AR*; rs2293275 for *LHCGR*; rs1062577 for *ESR1*; and rs1256049 for *ESR2* (S1 Table). SNPs in these genes were selected using *The National Center for Biotechnology Information* website [13].

DNA genotyping was performed using TaqMan® Genotyping Master Mix (Applied Biosystems, USA) which included all essential components (except probes, templates and water) for polymerase chain reaction (PCR). Allelic discrimination assays were carried out in a 7900HT Fast Real-Time PCR System (Applied Biosystems, USA). Results were read using SDS software v.2.4 (Applied Biosystems, USA). In order to warrant the results of genotyping we carried out a validation assay by Sanger sequencing (S2 Table). The validation cohort comprised a 3% of the genotyped samples (randomly selected) for each single SNP.

### Statistical analysis

Software package SPSS v.22 was used for statistical analyses (IBM Corporation, USA). The analyses included chi-square and Fisher exact tests for small samples size. The association between clinical variables and SNPs were analyzed by a binary logistic regression using different genetic models. Binary logistic regression was adjusted for PSA levels at diagnosis time, Gleason score, T stage and/or age. The biochemical recurrence free survival (BRFS) interval was estimated with Kaplan–Meier analysis and significance was determined by log-rank test. Cox regression analysis was used to assess the association of genetic variants and BRFS adjusting for PSA level at diagnosis, Gleason score, T stage and age Statistical signification was considered with p values ≤ 0.05. Genotypes analyses as well as Hardy–Weinberg equilibrium and Linkage disequilibrium (LD) analyses were performed using the online SNPStats software [14]. SNPs were in LD when they had a value of r^2^ > 0.5.

## Results

Nine SNPs across *AR*, *CYP17A1*, *LHCGR*, *ESR1* and *ESR2* genes were successfully genotyped in the total cohort (n = 311).

In the case of rs9332969 (AR gene is in X chromosome), all individuals were hemizygous for the *G* allele so it was discarded for later analyses.

Hardy-Weinberg analysis showed that all controls and patients were in equilibrium except for rs6152 (*AR* gene is in X chromosome). LD analysis showed a strong linkage between rs743572, rs6162 and rs6163, all of them in *CYP17A1* gene (S3 Table).

### Case-control study

Firstly, analysis was conducted as a case/control study, but none of the selected SNPs showed significant differences between PCa-confirmed patients and controls (data not shown). Genotyping and allelic distribution in this cohort is shown in Table 2.

**Table 2:** Genotyping and Allelic Proportion of SNPs.

SNP		n			Allelic Proportion	
		A*		G*	***A***	***G***
**rs6152 (*AR*)**	PCa	29	-	127	0.19	0.81
	Control	25	-	130	0.16	0.84
		A*		G*	***A***	***G***
**rs9332969**	PCa	-	-	156	0.00	1.00
**(*AR*)**	Control	-	-	155	0.00	1.00
		***AA***	***AG***	***GG***	***A***	***G***
**rs743572 (*CYP17A1*)**	PCa	67	66	23	0.64	0.36
	Control	53	82	20	0.61	0.39
		***AA***	***AG***	***GG***	***A***	***G***
**rs6162**	PCa	21	76	59	0.38	0.62
**(*CYP17A*1)**	Control	21	84	50	0.41	0.59
		***AA***	***AC***	***CC***	***A***	***C***
**rs6163 (*CYP17A1*)**	PCa	22	71	63	0.37	0.63
	Control	19	81	55	0.38	0.62
		***AA***	***AG***	***GG***	***A***	***G***
**rs1004467 (*CYP17A1*)**	PCa	127	28	1	0.9	0.1
	Control	114	39	2	0.86	0.14
		***CC***	***CT***	***TT***	***C***	***T***
**rs2293275**	PCa	63	78	15	0.65	0.35
**(*LHCGR*)**	Control	63	73	19	0.64	0.36
		***AA***	***AT***	***TT***	***A***	***T***
**rs1062577**	PCa	4	36	118	0.13	0.87
**(*ESR1*)**	Control	2	36	115	0.14	0.86
		***CC***	***CT***	TT**	***C***	***T***
**rs1256049**	PCa	139	17	-	0.95	0.05
**(*ESR2*)**	Control	141	14	-	0.95	0.05

* Men are hemizygous for rs6152 and rs9332969, because these SNP are in *AR* gene which is located on X chromosome.

** There are not *TT* carriers in the Iberian population. p-values are not included due to none of them reach significant values.

### Case-case study

#### Analysis of clinical variables

Secondly, we focused the analysis in PCa-confirmed patient population. We found statistically significant data in relation to PSA values (4-10ng/ml; 10-20ng/ml and >20 ng/ml) in several SNPs of the *CYP17A1* gene, such as rs743572 (p = 0.003); rs6162 (p = 0.015) and rs6163 (p = 0.010). Furthermore, we observed that the presence of *A* allele in rs743572 (p = 0.032; OR = 3.017, 95% CI (1.102–8.260); and the presence of *G* allele in rs6162 (p = 0.036; OR = 3.129; 95% CI (1.076–9.103) presented an increased risk of PSA values above 10 ng/ml versus *GG* and *AA* homozygous individuals, respectively. Concerning to Gleason score, we did not find any marker with statistically significant value, though rs1256049 in ESR2 gene was close to significance (p = 0.076) (S1 Fig). Regarding T stage, CT patients for rs2293275 in *LHCGR* gene were associated with more advanced stages, T3 and 4 (p = 0.037). See details in Tables 3 and 4.

**Table 3:** Significant associations between clinical variables and SNPs in PCa patients.

SNP	Genotypes	Clinical Variable			P-value*
		**PSA (ng/ml)**			
		4–10	10.1–20	> 20	
rs743572	*AA*	42	13	12	0.003
	*AG*	25	20	21	
	*GG*	17	0	6	
rs6162	*AA*	16	0	5	0.015
	*AG*	32	21	23	
	*GG*	36	12	11	
rs6163	*AA*	16	0	6	0.010
	*AC*	29	20	22	
	*CC*	39	13	11	
		**T Stage**			
		T1-T2	T3-T4		
rs2293275	*CC*	57	1		0.037
	*CT*	66	9		
	*TT*	14	0		
		**D´AmicoRisk**			
		Low	Intermediate	High	
rs743572	*AA*	33	17	15	0.008
	*AG*	13	28	24	
	*GG*	8	9	6	
rs6162	*AA*	8	8	5	0.019
	*AG*	17	32	26	
	*GG*	29	14	14	
rs6163	*AA*	8	8	6	0.015
	*AC*	15	30	25	
	*CC*	31	16	14	
rs1256049	*CC*	49	44	44	0.029
	*CT*	5	10	1	
	*TT*	0	0	0	

* Chi-square test.

Statistical not significant p-values not shown.

**Table 4:** Multivariate analysis for differentially distributed SNPs and clinical variables.

Clinical Variable	n	SNP	Dominant model			Recessive model		
				OR (95% CI)	p-value		OR (95% CI)	p-value
**PSA**, 4 < 10 vs. > 10 < 20 + > 20	156	rs743572	*AA vs*. *AG+GG*	1.871 (0.973–3.597)	0.060	*GG vs*. *AG+AA*	3.017 (1.102–8.260)	**0.032**
		rs6162	*GG vs*. *AG+AA*	1.603 (0.825–3.117)	0.164	*AA vs*. *AG+GG*	3.129 (1.076–9.103)	**0.036**
		rs6163	*CC vs*. *AC+AA*	1.731 (0.896–3.343)	0.103	*AA vs*. *AC+GG*	2.702 (0.984–7.417)	0.054
**T Stage**, 1–2 vs. 3–4	147	rs2293275	*CC vs*. *CT+TT*	7.392 (0.897–60.904)	0.063	*TT vs*. *CT+CC*	NA	NA
**D´Amico Risk**, low vs intermediate + high	153	rs743572	*AA vs*. *AG+GG*	3.856 (1.281–11.603)	**0.016**	*GG vs*. *AG+AA*	0.298 (0.086–1.033)	0.056
		rs6162	*GG vs*. *AG+AA*	3.574 (1.117–11.439)	**0.032**	*AA vs*. *AG+GG*	2.437 (0.686–8.659)	0.168
		rs6163	*CC vs*. *AC+AA*	3.866 (1.248–11.981)	**0.019**	*AA vs*. *AC+GG*	0.367 (0.105–1.290)	0.118
		rs1256049	*CC vs*. *CT+TT*	1.493 (0.352–6.331)	0.586	*TT vs*. *CT+CC*	NA*	NA*

* There is not *TT* carriers for rs1256049. Statistical test: logistic regression. PSA and T Stage analyses were adjusted for age. D´Amico Risk analyses were adjusted for age, PSA level at diagnostic, Gleason Score and T Stage. vs. versus, OR odds ratio, CI confidence interval, NA not applicable. As can be seen in column 2, n values are variable due to several patients are lost during the follow up.

We found statistical associations between D´Amico risk classification and the following SNPs in the *CYP17A1* gene: rs743572 (p = 0.008), rs6162 (p = 0.019), rs6163 (p = 0.015), and rs1256049 (p = 0.029) (Table 3). Moreover *G* allele presence in rs743572 confers an increased risk of being in intermediate and high risk stratification versus *AA* patients (p = 0.016; OR = 3.856; 95% CI (1.281–11.603)). The presence of *A* allele in rs6162 confers an increased risk of being in intermediate and high risk stratification versus *GG* (p = 0.032; OR = 3.574; 95% CI (1.117–11.439)); and in rs6163 *A* allele confers an increased risk of being in intermediate and high risk stratification versus *CC* (p = 0.019; OR = 3.866; 95% CI (1.248–11.981)) (Table 4).

#### Analysis of biochemical recurrence

A total of 148 patients were included in the biochemical recurrence analysis after initiation of primary treatment. Of them, 32 (21.6%) received ADT (androgen deprivation therapy); 73 (49.3%) and 38 (25.7%) patients underwent radiotherapy and radical prostatectomy, respectively, and only 5 patients (3.4%) remained in active surveillance. In general, within 54 months of observation after treatment, 123 (83.1%) patients did not show significant increases in PSA levels and 25 (16.9%) of them presented a biochemical recurrence.

After stratifying by the type of therapy received, we observed that the rate of biochemical recurrence was 31.3% for patients treated with ADT, 6.8% for those treated with radiotherapy and 26.3% for patients undergoing radical prostatectomy. None of the patients who remained in active surveillance manifested biochemical recurrence.

**Fig 1:**
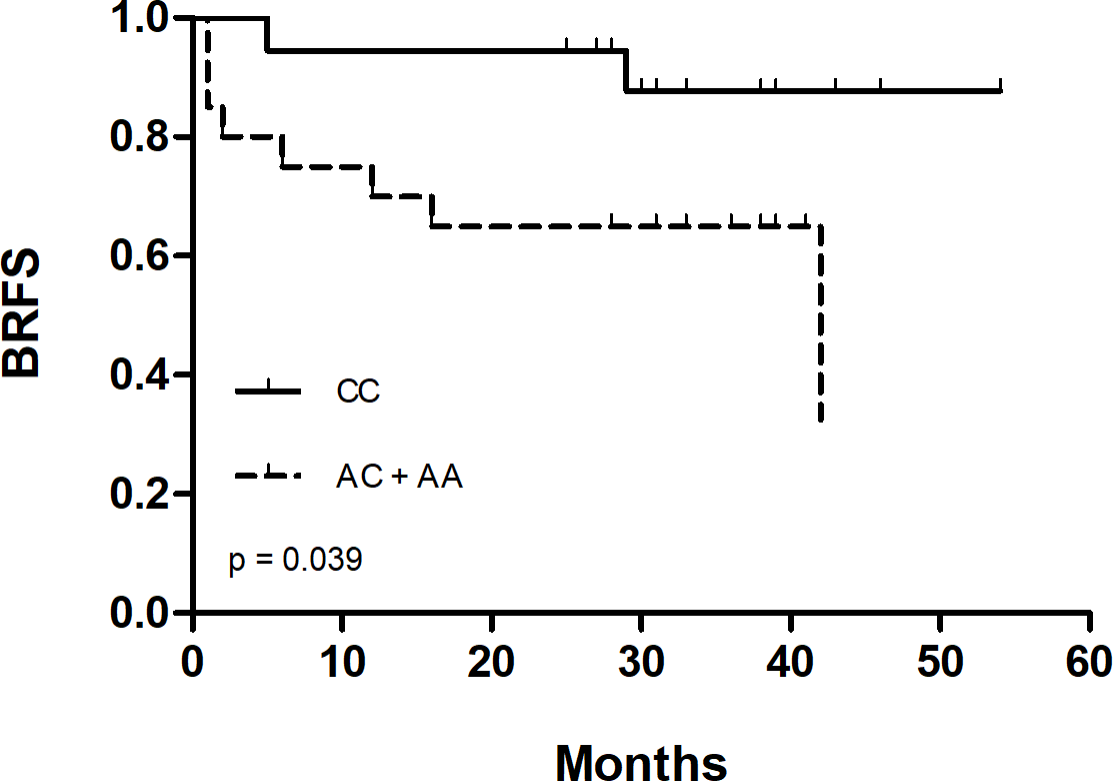
BRFS according rs6163 (*CYP17A1*) genotype. Kaplan-Meier curves of time to biochemical recurrence in patients treated with radical prostatectomy and stratified by rs6163 genotype (*CC vs*. *AC+AA*). P value obtained from log-rank t test.

As SNPs rs743572, rs6162 and rs6163 showed an increased risk of D´Amico risk, we performed a Kaplan Meier analysis and log-rank test for ADT, radiotherapy and radical prostatectomy therapies. For ADT as well as for radiotherapy, none of the SNPs were significantly associated to BRFS. However, for radical prostatectomy *AC* and *AA* genotypes in rs6163 (*CYPI7A1*) were significantly associated with a shorter BRFS compared to the *CC* genotype (log-rank p = 0.039), 29.10 months vs 49.59 months, respectively (Fig 1). When a Cox regression multivariable analysis was performed, rs6163 was not an independent variable for risk to BRFS after radical prostatectomy (p = 0. 221; OR = 2.822; 95% CI (0.535–14.885)).

#### Multigene analysis

Multigene analysis of SNPs revealed that the genotype GTGTAACA for variants rs743572, rs2293275, rs6152, rs1062577, rs6162, rs6163, rs1256049 and rs1004467 (in this order) was associated with significant increased risk according to D´Amico classification (p = 0.0045; OR: 0.6, 95% CI(0.19–1.02)); higher PSA values (p = 0.0011; OR: 0.68, 95% CI (0.35–1.31)); and increased risk of Gleason score ≥7 (p = 0.026; OR: 5.21, 95% CI (1.23–22.02)). Regarding T stage, patients with genotype GCGTAACA (rs743572, rs2293275, rs6152, rs1062577, rs6162, rs6163, rs1256049 and rs1004467, in this order) showed an increased risk of having higher T stage values such as T3 and T4 (p = 0.045; OR: 6.34, 95% CI (1.06–37.88)) (S4 Table).

Conversely, TAAG genotype for SNPs rs1062577, rs6162, rs6163 and rs1004467 seem to protect from higher PSA values (p = 0.016; OR: - 0.43, 95% CI (-0.79–-0.08)) and ATGGCCA genotype for SNPs rs743572, rs2293275, rs6152, rs6162, rs6163, rs1256049 and rs1004467 showed a protective effect for higher Gleason scores (values ≥7) (p = <0.0001; OR: -0.38, 95% CI (-0.37–-0.40)). No genotypes were significantly associated with risk of PCa (S4 Table).

## Discussion

PCa is a heterogeneous disease as evidenced by its variable clinical course [15]. PSA level, core biopsies, T stage and Gleason scores used for initial evaluation offer limited information to clinicians to determine diagnosis and harshness of disease [16]. Current clinical risk groups, used in clinical routine, seem to misclassify patients leading to over/undertreatment of these patients [16]. New and reliable tools are needed to improve the precision in diagnosis and stratification of PCa patients, and genetic markers could be the most suitable ones. In the last decade, development of high throughput technologies have favored the identification of genetic variations associated with PCa and their incorporation into clinical practice offers an opportunity to ease clinical decisions [17].

There is scarce data in relation to genetic germline biomarkers for PCa prognosis and stratification. Main researches are focused on *RNAseL* (locus HPC1), *ELAC2* (locus HPC2), *MSR1* (chromosome 8) [18] and *BRCA1/2* genes [19], but no conclusive data have been reported. For instance, it is known that PCa patients harboring germline DNA repair alterations generally have a worse clinical evolution or earlier cancer events. However, little is known of a genetic stratification of the disease or even genetic responsiveness to radical prostatectomy, radiotherapy or hormonal therapies including ADT and second-generation hormonal agents (such as abiraterone and enzalutamide) [20]. PCa is a hormone-dependent cancer; the androgen receptor (*AR*) axis plays a pivotal role in both disease development and progression [21]; for this reason we focused on PCa sex hormones related genes, to perform a retrospective study for the relation among *AR*, *CYP17A1*, *LHCGR* and *ESR* polymorphisms with PCa predisposition and severity.

We could not prove any statistically significant difference between controls and patients but when the analysis was focused on PCa patients we proved these genetic markers had a predictive role on PCa aggressiveness characteristics (PSA, D´Amico risk and T stages). It is known that any change in androgen synthesis and metabolism genes can strongly affect the progression of PCa and the response to treatments [22]. Our aim is to try to discover optimal biomarkers associated to PCa aggressiveness such as recent patented genes like rs4054823 (17p12.) [23, 24].

Recent studies of NGS or Exome sequencing focused on finding new PCa biomarkers, have found that rs33999879 (SMC4) was a predictor for Gleason scores upgrade [25]. These NGS data also found that carrying any mutations at pathogenic germline variants (*ATM*, *ATR*, *BRCA2*, *FANCL*, *MSR1*, *MUTYH*, *RB1*, *TSHR* and *WRN*) were frequently observed in patients with metastatic CRPC (castration-resistant PCa) [26]. However, no data yet had analyzed the role of *AR*, *CYP17A1*, *LHCGR* and *ESR* genes in PCa.

For *CYP17A1* gene we found that several SNPs (rs743572, rs6162 and rs6163) were statistically associated with a more aggressive PCa (PSA values > 10 ng/ml and a higher D´Amico risk). *CYP17A1* gene encodes a key enzyme in the steroidogenic pathway that produces progestins, mineralocorticoids, glucocorticoids, androgens, and estrogens, and it has a critical function in PCa [27]. There is no published data that had previously established any relationship between clinical stages and *CYP17A1* SNPs in African ancestry or Caucasian populations [28].

*In silico* analyses for *CYP17A1* variants have showed that rs1004467 is on intronic region, rs743572 is on 5´ untranslated region; and rs6162 and rs6163, are both coding-synonymous (H46H and S65S, respectively) and with a tolerated phenotypic effect [29] (SITF score: 0.31 and 1, respectively). The presence of *CYP17A1* SNPs is further associated with altered levels of circulating DHEA-S (dehydroepiandrosterone sulfate) in Caucasians, which likely modify steroid precursor levels available for intracrine conversion to more potent hormones in tissues and prostatic cells [7]. In addition, genetic polymorphisms in the *CYP17A1* gene have been significantly associated with a risk of progression to CRPC [30], but there are no details on risk stratification. A recent meta-analysis carried out by Wang et al, did not find any significant association between rs743572 polymorphism and PCa risk but it was suggested that *CYP17A1* rs743572 might modify the risk of PCa in the individuals of African origin [31].

In the present study only rs6163 showed a significant association with a shorter BRFS in patients with prostatectomy. There is not much data about genetic markers in androgen metabolism as indicators of outcomes after PCa therapies and scarce data in relation to *CYP17A1* gene [7, 20]. Wright et al. investigated the genetic association between three SNPs in *CYP17A1* (rs743572, rs10883783 and rs17115100) and their responses to treatment (principally prostatectomy), but they did not find any differences in the risk of recurrence/progression by this genotype analysis [32]. However SNPs in other genes such as *SRD5A* and *HSD17B*, both involved in androgen metabolism, were shown to be associated to biochemical recurrence in Caucasian and Asian PCa patients after prostatectomy [22, 33]. rs1004467 (*CYP17A1*) was previously found to be associated with PCa risk and disease progression after ADT among Japanese population [30]. It is proved that serum dihidrotestosterone (DHT) level and testosterone was significantly elevated in *GG* genotype for rs1004467 (*CYP17A1*) compared to *A* allele carriers [34]. Studies have shown that those individuals with higher levels of DHT were susceptible to CRPC development in the future. This could provide insights for an early PCa detection, diagnosis, management and potential therapeutic targets [34]. Last, our LD analysis showed a strong linkage between rs743572, rs6162 and rs6163, (*CYP17A1*). Similar results were supported by other studies proving that both, rs6162 and rs6163 were strongly linked with rs743572, so these SNPs exhibit their functions in coordination with rs743572 as a risk genotype [30].

In relation to *ESR* genes, we demonstrated a statistical association between rs1256049 (*ESR2*) and a higher D´Amico risk. Several studies have focused on the effect of *ESR* polymorphisms (like rs9340799 and rs1256049) on PCa development, but none of them have evaluated their relation to PCa aggressiveness. rs1256049 is a silent mutation in codon 328 and *G*>*A* change has a direct effect on modifying the secondary structure of the mRNA; leading to changes in mRNA stability and translation which makes it as a candidate polymorphism for PCa susceptibility in Caucasians [35, 36]. This SNP has been previously associated to an increased PCa risk, both in codominant and recessive genetic models [35].

Despite, *ESR1* (rs1062577) was not associated with any clinical variable in the present study, it was previously related to changes in plasma steroid levels conducting cancer aggressiveness and efficacy of ADT [7]. We have proved with an *in silico* analysis that *A* allele (rs1062577) generates a new miRNA binding site for hsa-miR-3646, hsa-miR-3662 and hsa-miR-5585-3p [37]. In PCa there are similar expression patterns for miR-3646 and miR-3662, similarly happens in other hormonal dependant tumors like breast, ovarian or uterine [37]. For example, in breast cancer, miR-3646 produces cellular proliferation [38]. Moreover A allele in rs1062577 (*ESR1*) produces a loss of miR-186 and miR-6507-5p binding site [37]. miR-186 has a role as a PCa tumor suppressor, so any alteration on this SNP will be related to an oncological event [39].

We demonstrated in *LHCGR* gene, a relation between rs2293275 and high T stages. Other researchers had found an association between PCa incidence and this variant in different racial groups by no for stage of the disease [40]. rs2293275 produces a missense mutation with an amino acid change (N312S) [29]. Position 312 is located in exon 10 and this is important for receptor activation [40], but this amino acid change seems to not affect frameshift region so it has a tolerated phenotypic effect (SIFT score: 0.774) [29].

Finally, we did not find any significant association with PCa for *AR* SNPs (rs6152 and rs9332969). Likewise, a previous study in a Caucasian population did not find differences between case-control for rs6152 but it was described an association for this polymorphism with advances stages [41]. rs6152 is a coding-synonymous region (E213E) not frameshift alteration and with a tolerated phenotypic effect [29] (SITF score: 1). In relation to rs9332969, despite we not report any allelic variation (100% of patients and controls are *G* carriers), this SNP seems to be an attractive candidate biomarker for PCa aggressiveness because is a missense variant with an amino acid change (R841H) [29]. R841H variation causes androgen insensitivity syndrome [42]. This is due because *AR* protein with R841H variation alters the interaction with androgens which result in a partial *AR* functional disruption [42] and its frequency and effect had not been previously studied in the European population [43].

We fully acknowledge that our results must be interpreted with caution, as the sample size is limited. One core limitation of the study is the low specificity of prostate biopsy; although we tried to control this issue by performing systematic 20-core biopsies, the number of false negatives could still be high. Moreover, the observation period was relatively short (median follow-up of 34.18 month) and only one-third of the patients had a high risk of recurrence; and the number of patient in each treatment group is reduced. Nevertheless, this is the first time that the role of *AR*, *CYP17A1*, *LHCGR* and *ESR* polymorphisms have been studied in relation to PCa aggressiveness.

## Conclusion

We describe the initial roles of *CYP17A1*, *AR*, *LHCGR* and *ESR* as risk disease biomarkers. We believe that rs743572, rs6162, rs6163, and rs1256049 (*CYP17A1*); and rs2293275 (*LHCGR*) are promise biomarkers for PCa aggressiveness. Future studies seem warranted in order to evaluate the real predictive and prognostic impact of *CYP17A1*, *AR*, *LHCGR* and *ESR* variations on each specific treatment response in a larger cohort of patients within longer observation periods.

## Supporting information

S1 Fig Association between rs1256049 (*ESR2*) and Gleason score. (TIF)

S1 Table Description of SNPs included in the study. Information was obtained from Ensembl and dbSNP data base, National Center for Biotechnology Information (NCBI).
(XLSX)

S2 Table Primers sequence details. (XLSX)

S3 Table Significant linkage disequilibrium for variants. Test of linkage disequilibrium for all pairs of loci performed with SNPstats software. In blue, all polymorphism that are out of balance (significance level = 0.05). p = p-value; r2 = standardized disequilibrium values.
(XLSX)

S4 Table Multigene analysis. All genotypes with a frequency less than 5% in the population have been omitted.
(XLSX)
