# Supplementary figures and images for: Association between polymorphisms in sex hormones synthesis and metabolism and prostate cancer aggressiveness
Source: PLoS One. 2017 Oct 5;12(10):e0185447. doi: 10.1371/journal.pone.0185447 (PMC5628818; doi:10.1371/journal.pone.0185447)

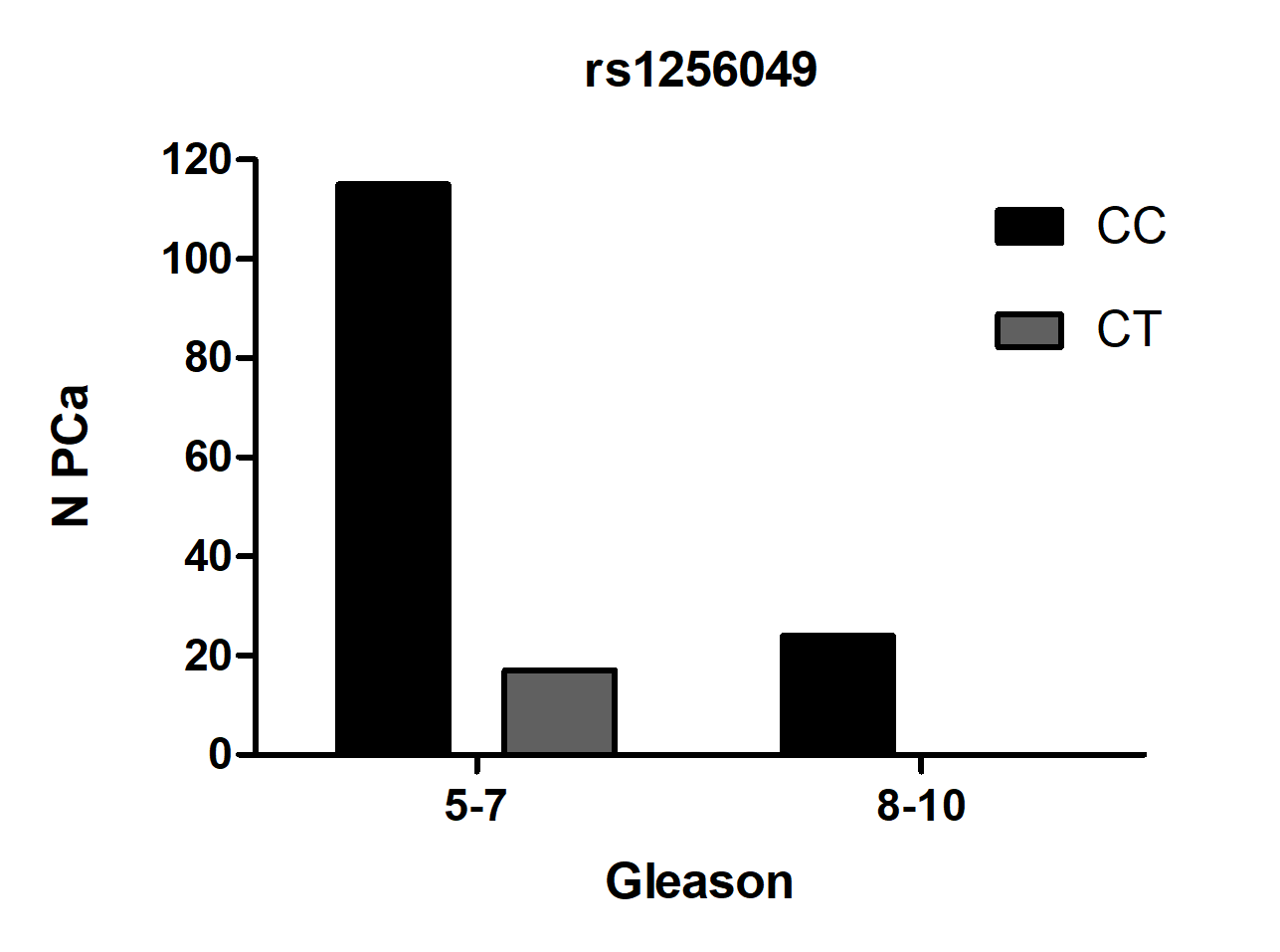

Supplement: S1 Fig — (TIF) [file pone.0185447.s001.tif]
